# Polycyclic aromatic hydrocarbons, gut microbiome composition, impulsivity, and attention covary in a human cohort

**DOI:** 10.1128/spectrum.00783-26

**Published:** 2026-09-21

**Authors:** Austin J. Hammer, Kristin D. Kasschau, Edward W. Davis II, Peter Hoffman, Katie Johnson-Camacho, Alexandra Alexiev, Kim Anderson, Jackilen Shannon, Lisa K. Marriott, Thomas J. Sharpton

**Affiliations:** 1 Department of Microbiology, Oregon State University 2694, Corvallis, Oregon, USA; 2 Center for Quantitative Life Sciences, Oregon State University 2694, Corvallis, Oregon, USA; 3 Department of Environmental and Molecular Toxicology, Oregon State University 2694, Corvallis, Oregon, USA; 4 Department of Medicine, University of California Los Angeles 8783, Los Angeles, California, USA; 5 Knight Cancer Institute 608031, Portland, Oregon, USA; 6 School of Medicine, Oregon Health & Science University 89020, Portland, Oregon, USA; 7 School of Public Health, Oregon Health & Science University 6684 , Portland, Oregon, USA; 8 Department of Statistics, Oregon State University 2694, Corvallis, Oregon, USA; 9 Linus Pauling Institute, Oregon State University 2694, Corvallis, Oregon, USA; Cleveland Clinic Lerner Research Institute, Cleveland, Ohio, USA

**Keywords:** polycyclic aromatic hydrocarbons, human gut microbiome, impulsivity, attention, human behavior

## Abstract

Polycyclic aromatic hydrocarbons (PAHs) are pervasive environmental pollutants linked to adverse neurobehavioral outcomes, yet the biological pathways coupling exposure to behavior are poorly defined. The gut microbiome is both sensitive to PAH exposure and a modulator of central nervous system function, suggesting it may mediate how PAH exposure influences neurobehavior. We tested whether PAH exposure, gut microbiome composition, and neurobehavioral function covary in a statewide sample of 34 adults stratified into high-impulsivity/poor-attention (HH) and low-impulsivity/fast-attention (LL) groups. Participants wore silicone wristbands for 30 days to passively sample PAH exposure and provided a single fecal sample for 16S rRNA profiling at the end of the wear period. Higher PAH exposure was associated with HH group membership in a sex-dependent manner, with the largest elevations among HH males. At the community level, PAH exposure profiles correlated with microbiome dissimilarity, and HH membership was associated with increased alpha-diversity and altered community composition relative to LL members. At the taxon level, 22 genera were significantly associated with 14 PAH compounds (FDR < 0.1). No individual genera were significantly associated with neurobehavioral group after multiple testing correction. Nevertheless, cross-referencing PAH-responsive genera (FDR < 0.1) against those with nominal neurobehavioral associations (*P* < 0.05) identified two candidate genera*—Hydrogenoanaerobacterium* and *Methanobrevibacter*—whose abundance covaries with both PAH exposure and neurobehavioral phenotype. Both have been independently linked to cognitive or neurological outcomes in prior work. These findings support a three-way relationship among environmental chemical exposure, gut microbiome composition, and neurobehavioral function, establishing an empirical foundation for testing microbiome-mediated links between PAH exposure and neurobehavioral outcomes.

**IMPORTANCE:**

Polycyclic aromatic hydrocarbon (PAH) exposure is widespread and is associated with impulsivity and attention problems, but how exposure translates into neurobehavioral risk is unclear. The gut microbiome is a plausible intermediary: gut microbes biotransform environmental chemicals and produce metabolites that influence brain function. In a statewide adult cohort, higher PAH exposure associated with with greater impulsivity and poorer attention in a sex-dependent manner, and both PAH exposure and neurobehavioral phenotype were associated with distinct gut microbiome features at the community and taxon levels. We identify candidate genera at the intersection of PAH exposure and neurobehavioral group whose biology independently implicates them in cognitive and neurological function. Because their associations with neurobehavioral phenotype are nominal, we present these genera as hypothesis-generating candidates for future study. By showing that all three domains covary within a single cohort, this work moves beyond pairwise associations toward testable microbiome-targeted and exposure-reduction strategies for PAH-related neurobehavioral risk.

## INTRODUCTION

Polycyclic aromatic hydrocarbons (PAHs) are a group of persistent and ubiquitous environmental pollutants that are principally produced during incomplete combustion of organic matter, such as during the burning of fossil fuels and biomass (1–3). These persistent organic pollutants can linger in a variety of environmental locales, such as air, water, soil, and food, and are known to bioaccumulate in organisms (4–7). PAHs readily cross the blood–brain barrier due to their lipophilic nature (8) and exert especially toxic effects on the brain and neural tissue, where they are linked to increased susceptibility to cognitive and neurological disorders (9–11). Research investigating prenatal and early postnatal exposure to PAHs has consistently reported negative impacts on childhood behavioral development, including hyperactivity and attention deficits (12–15). Higher levels of PAH exposure in children correlate with increased inattentiveness and hyperactivity (12–14), and exposure to PAHs and other xenobiotics correlates with impulsive behavior in both children and adults (13, 16, 17). Notably, PAH metabolism shows pronounced sex-dependent variation (18, 19), suggesting that the neurobehavioral consequences of exposure may differ between males and females. Despite clear links between PAH exposure and neurobehavioral outcomes, the intermediate biological systems that connect exposure to behavioral change remain poorly characterized, particularly in humans (20, 21).

The gut microbiome is emerging as a plausible intermediary in this pathway. Recent research demonstrates that the gut microbiome is sensitive to PAH exposure and may play a critical role in mediating the toxicity of xenobiotic compounds, including PAHs (22, 23). Gut microorganisms possess the enzymatic capacity to metabolize PAHs, and this metabolism can produce both toxic (24) and non-toxic byproducts (25, 26) that exert their own effects on host physiology (27), including bioactive compounds such as estrogenic metabolites (28, 29). Candidate routes for this biotransformation include the direct hydroxylation to estrogenic metabolites noted above, deconjugation of host-conjugated metabolites by microbial beta-glucuronidases (30), and cytochrome P450-type oxidation, although the largely anaerobic gut lumen may favor reductive transformations. Many gut taxa display altered abundance following broad PAH exposure (31, 32), and studies in animal models demonstrate that exposure to the PAH benzo[a]pyrene (BaP) shifts the overall composition of the gut microbiota (33–36). Critically, in zebrafish, the microbiome has been shown to mediate the impact of BaP on neurodevelopment (36), providing direct evidence that gut microbes can modulate PAH-driven neurobehavioral outcomes. Independent of PAH exposure, gut microbiome composition is linked to behavioral variation in human clinical studies (37–39), and gut microbes modulate production of neurologically relevant metabolites, including short-chain fatty acids and neurotransmitters such as serotonin, that influence neural function (40–42). Taken together, these lines of evidence support the hypothesis that the gut microbiome is a biological intermediary between environmental PAH exposure and neurobehavioral outcomes, through its capacity to biotransform PAH compounds into bioactive metabolites, its sensitivity to PAH-induced compositional disruption, and its established influence on brain function and behavior.

Prior work has established pairwise links between PAH exposure and the gut microbiome, PAH exposure and impulsivity/attention, and the microbiome and behavior, but these three factors have not been examined simultaneously within a single cohort. Our study addresses this gap by testing whether PAH exposure, gut microbiome composition, impulsivity, and attention converge in humans. Adults were recruited from a large statewide population-based cohort study (43, 44) into the Exposure, Microbiome, and Cognition Cohort (EMC2), stratified into groups with concordantly high impulsivity/poor attention (HH) or concordantly low impulsivity/fast attention (LL). We integrated passively sampled PAH exposure profiles from silicone wristbands, 16S rRNA gut microbiome profiles, and neurobehavioral assessments to jointly examine the relationship between PAH exposure, gut microbiome composition, and neurobehavior in 34 participants with complete paired data. Overall, our analyses find that higher PAH exposure is associated with HH group membership in a sex-dependent manner, that both PAH exposure and neurobehavioral group track with shifts in gut microbiome composition, and that specific microbial genera respond to PAH exposure at the taxon level. Together, these results provide evidence that environmental chemical exposure, the gut microbiome, and neurobehavioral function form an interacting system within a single human cohort.

## MATERIALS AND METHODS

### Study context and setting

This multi-site cross-sectional study engaged adults in an EMC2 (OHSU IRB study #23221, eCRIS #6688). The EMC2 study leveraged established research infrastructure from the Healthy Oregon Project (HOP, OHSU IRB #18473), which afforded access to a large, geographically distributed population that was already familiar with and adherent to clinical study practices. The Healthy Oregon Project (www.healthyoregonproject.org) is an Oregon-wide population-based cohort that supports basic and clinical research, and importantly, aims to influence the health of Oregonians through providing no-cost genetic screening for heritable cancers (43). HOP employs a cost-effective remote recruitment approach that establishes a large-scale cohort for population-based studies (44).

### EMC2 study design for participant recruitment

The EMC2 pilot study leveraged HOP’s research infrastructure for participant recruitment, selection, and data collection in compliance with both IRB-approved protocols. Due to budgetary limits, a sample of 50 individuals was recruited for this pilot study by email, 34 of whom provided complete paired data across all three domains (fecal microbiome, wristband PAH exposure, and neurobehavioral assessment; Table S1). Participant flow through recruitment and selection is shown in Fig. S1. Targeted study recruitment for cases and controls leveraged neurobehavioral data to identify the highest (HH) and lowest (LL) quartiles of impulsivity and attention. Participants in HH or LL recruitment groups were emailed by the HOP study team about the study opportunity. EMC2 participants consented via HOP app to provide a fecal sample for microbiome analysis and submit their worn silicone-based wristbands to quantify participants’ passively accumulated PAHs from the environment. This study’s eligibility criteria excluded individuals with antibiotic use in the past 6 months. Inclusion criteria were 18 years of age or older, consent to the HOP repository, creation of a secure Medable account, completion of study behavioral assessments, and consent to this study.

### Behavioral assessments

All HOP participants were invited to complete data collection modules that assess lifestyle behaviors and health risks (44), including neurobehavioral measures of impulsivity and attention used to select participants for the EMC2 study. Quartiles for each measure identified potential participants for case–control groups.

#### Impulsivity

HOP participants completed the Barratt Impulsiveness Scale (BIS-15) (45), a 15-item instrument scored on a 4-point Likert scale to assess total impulsivity in clinical and non-clinical populations (46). Published work from our team details BIS-15 implementation with adult (47) and adolescent (48) populations. BIS-15 includes attentional, motor, and non-planning facets of impulsivity, with total scores ranging from 15 to 60. Quartiles applied previously defined cut points for Q1 (15–28, least impulsive), Q2 (29–33), Q3 (34–37), and Q4 (38–60, most impulsive) to data from 3,106 HOP participants who completed BIS-15 at the time of EMC2 recruitment (Table S2).

#### Attention

Participants completed a validated 3-min version of the Psychomotor Vigilance Task (PVT) (49), termed PVT-B (50). PVT-B provides an objective measure of reaction time (RT), errors of omission (i.e., lapses and lack of response in sufficient time), and errors of commission (i.e., false starts, where participants provide a response without a stimulus). At the time of EMC2 recruitment, 2,678 participants completed PVT-B, with individuals’ median reaction time used to assign quartile cut points. Individuals with RTs >1,300 ms were excluded as outliers (*n* = 3, 2–16× higher than the remaining data), with quartiles comprising Q1 (≤14 ms, lowest and fastest reaction times), Q2 (315–339 ms), Q3 (340–370 ms), and Q4 (≥371 ms, longest and slowest reaction times; Table S2).

#### Defining case–control groups for recruitment

Cases were defined as participants in the highest quartiles (Q4) for both impulsivity and attention, termed high/high (HH) to refer to individuals with high impulsivity and long reaction times. Controls in this study represented the lowest quartile (Q1), designated as low/low (LL), to refer to participants with low impulsivity and low (i.e., fast) reaction times. A total of 2,628 participants completed both instruments, with quartile distributions shown in Table S2, with no difference between group sizes (chi-square *P* = 0.33). Once removing participants who were missing sex, age, impulsivity data, or reaction times, a total of 422 participants qualified for LL or HH group recruitment, with ages comparable between groups (*P* = 0.77; LL = 40.4 years ± 11.8 SD; HH = 40.8 ± 12.0). Controls (LL = 283, 79.5% female) and cases (HH = 139, 85.6% female) had similar sex distributions (*P* = 0.12). The relationship between impulsivity and attention in the EMC2 recruitment sample was confirmed by independent sample *t*-tests, with significant differences observed between impulsivity quartiles (i.e., Q1 vs Q4) for all attention metrics studied (*P* < 0.005, Table S3). In addition to higher reaction times, participants in the most impulsive quartile (Q4) had more lapses and fewer false starts, consistent with less attention and slow response times. Thus, study controls represented the lowest quartiles (LL) of impulsivity and response latency (i.e., more attention), while the highest quartiles (HH) represented participants with high impulsivity and higher (i.e., slower) reaction times indicative of worse attention.

### Passive PAH exposure sampling using silicone wristbands

#### Preparation and deployment

To measure each participant’s PAH exposure, we prepared silicone wristbands (24hourwristbands.com, Houston, TX, USA) that enable passive sampling of 34 distinct PAHs (51). We rinsed wristbands with deionized water and used a vacuum oven (300°C for 12 h at 0.1 Torr; Blue M vacuum oven, no. POM18VC; Welch DuoSeal pump, no. 1405, Mt. Prospect, IL, USA) to remove manufacturing process-related chemicals in the silicone polymer. Clean wristbands were mailed to participants, who wore the wristbands for 30 consecutive days prior to stool sampling (below). Used wristbands were then sealed and shipped back to us. We collected and analyzed quality control samples from each group of prepared wristbands to ensure all wristbands met our data quality objectives (52).

#### Cleaning and extraction

We cleaned wristbands that met our inclusion criteria and extracted the chemicals from the silicone as previously described (53). Particles on the wristband surface do not reflect the chemical fraction available to be absorbed by the body, and we removed particles with two rinses of 18 MΩ·cm water and one rinse of isopropanol (51, 53). We extracted chemicals by adding two separate 50 mL volumes of ethyl acetate at room temperature to the wristbands. We quantitatively concentrated the ethyl acetate to 1 mL using TurboVap 500 Closed Cell Evaporators and a TurboVap LV evaporator workstation (Biotage LLC, Charlotte, NC, USA).

#### Chemical analysis

We quantitatively analyzed all wristband extracts for 64 PAHs and alkylated PAHs. Analysis was performed using an Agilent 7890B gas chromatograph and Agilent 7000C triple quadrupole mass spectrometer with a J&W Select PAH column (30 m × 0.25 mm × 0.15 µm). Deuterated surrogates were used as internal standards for targets with similar physicochemical properties. Instrument performance was verified at the beginning and end of each run and every 10–15 samples with a calibration check using Standard Reference Material 1991 (National Institute of Standards and Technology). Solvent blanks were analyzed every 10–15 samples and at the beginning and end of each run. Duplicate sample aliquots were analyzed every 15 samples. In all cases, checked standards, matrix spikes, and duplicate samples met laboratory data quality objectives (within 30% of nominal or relative percent difference less than 30%).

### Microbial 16S rRNA library preparation and sequencing

Fecal samples were self-collected by human participants using the OMNIgene GUT OM200 Stool Sampling Kit (DNA Genotek Inc, Ottawa, Ontario, Canada) and shipped to the Microbiome Core Facility at Oregon State University for downstream processing. Stool samples were frozen at −80°C prior to processing all samples for DNA extraction and 16S rRNA library preparation in a single batch following our prior work (54). Briefly, after a 10-min incubation, samples were subjected to bead beating. PCR was performed in triplicate using 1 μL of purified DNA from the lysis solution to amplify the V4 region of the 16S rRNA gene, using the 515F (5′-GTGYCAGCMGCCGCGGTAA-3′) and 806rB (5′-GGACTACNVGGGTWTCTAAT-3′) primer set (55). An equal quantity of DNA was selected from each of the samples, and the pooled collection of DNA was cleaned, then diluted and pooled prior to sequencing by the Center for Quantitative Life Sciences at Oregon State University, using a MiSeq instrument (Illumina, San Diego, CA, USA) with 300-bp paired-end reads.

### PAH and gut microbiome analyses

Using the targeted PAH exposure metabolite data set, we investigated the relationship between PAH exposure to covariates, including neurobehavioral group (HH/LL) and sex. Overdispersion was tested using a Poisson model of PAH abundance, evaluating PAHs and PAH metabolites, which displayed a dispersion parameter greater than 1. Differences in PAH exposure abundance were modeled as a function of HH vs LL neurobehavioral group, with negative binomial generalized linear models used due to high overdispersion among nearly all metabolites. *P* values from these models were adjusted using false discovery rate, selecting only metabolites that were associated with the neurobehavioral group below a predefined *q* value of 0.1. We also implemented redundancy analysis to perform direct gradient analysis, creating a distance matrix based on PAH abundance using the Euclidean distance metric. We subsequently tested the relationship between the neurobehavioral group and overall PAH exposure, considering sex-dependent effects, using PERMANOVA.

Gut microbiome sequences were filtered for read quality and clustered into amplicon sequence variants using DADA2 (56) with R 4.1.2 and taxonomically annotated using the SILVA reference annotation following our prior approach (57). Alpha- and beta-diversity analyses were performed using a relative abundance-normalized sequence count table. Generalized linear models were used to model species richness, phylogenetic richness, and Shannon diversity as a function of neurobehavioral group (HH vs LL). Unweighted UniFrac ordination axes were generated using vegan (58). To clarify how neurobehavioral group relates to gut community composition, ANOVA was applied to the capscale-generated model of unweighted UniFrac distances.

The correlation between the overall PAH exposure profile and the composition of the gut microbiome was assessed using a Mantel test with Spearman correlation (5,000 permutations), comparing pairwise Euclidean distances of standardized PAH concentrations against pairwise Bray–Curtis dissimilarities computed from rarefied 16S rRNA gene amplicon data. A linear model on the pairwise distance matrices was also fit as a parametric complement to the Mantel test.

To identify associations between individual microbial genera and PAH exposure and between microbial genera and neurobehavioral group, we used MaAsLin2 (Microbiome Multivariable Associations with Linear Models, v1.22.0) (59). Genus-level abundances were total-sum-scaled, normalized, and log-transformed prior to model fitting. For taxon–PAH associations, each genus was modeled as a function of scaled PAH concentration for each of 30 PAH compounds individually. For taxon–impulsivity associations, each genus was modeled as a function of cohort membership (HH vs LL). *P* values were adjusted using the Benjamini–Hochberg false discovery rate correction across all tests within each analysis, with significance defined at FDR < 0.1. Visualizations were created using ggplot2 (60).

## RESULTS

### PAH exposure is highly variable and overdispersed across the EMC2 cohort

**Fig 1:**
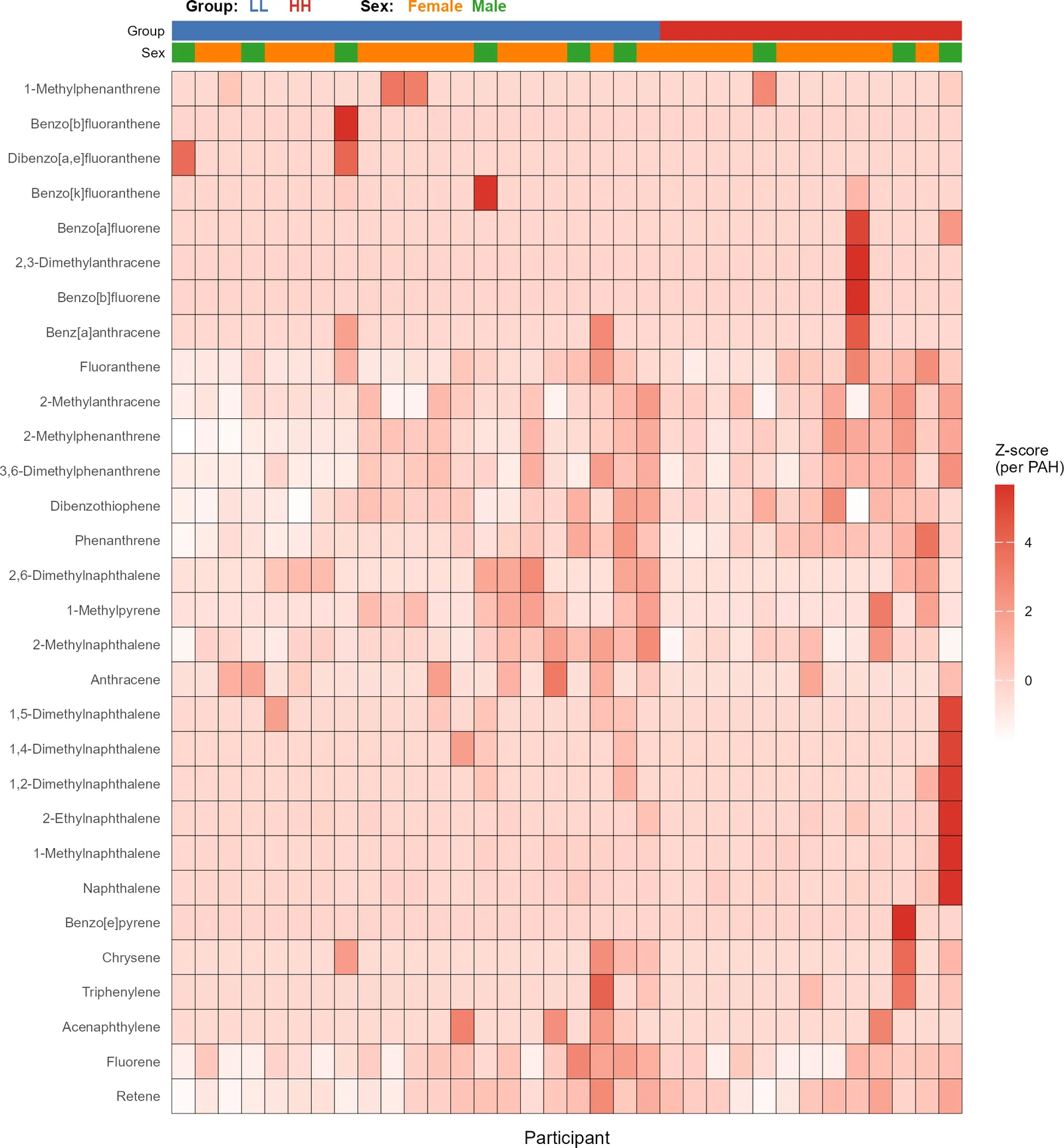
PAH exposure across the EMC2 cohort. Heatmap of 30 PAH compounds (rows) measured from silicone wristbands worn by 34 participants (columns). Values are *Z*-score normalized within each PAH compound, with darker shading indicating higher relative exposure. Participants are grouped by neurobehavioral classification (LL, low impulsivity/fast attention, blue; HH, high impulsivity/poor attention, red) and sex (female, orange; male, green), as indicated by the annotation bars.

Earlier work has demonstrated that PAH exposure is not uniform across human populations (61–64). Our findings are consistent with prior work testing the hypothesis that PAH exposure is highly variable and disproportionately experienced across cohort participants. PAHs vary in at least 10% of subjects, with more than 88% of the selected PAHs’ variance of exposure being much greater than the mean (GLM–Poisson, dispersion parameter >1). This variation is readily observed visually, where several individuals display exposure to PAHs, such as naphthalene, at two to three orders of magnitude greater levels than other subjects in these data (Fig. 1).

### Impulsivity and attention are linked to PAH exposure in a sex-dependent fashion

**Fig 2:**
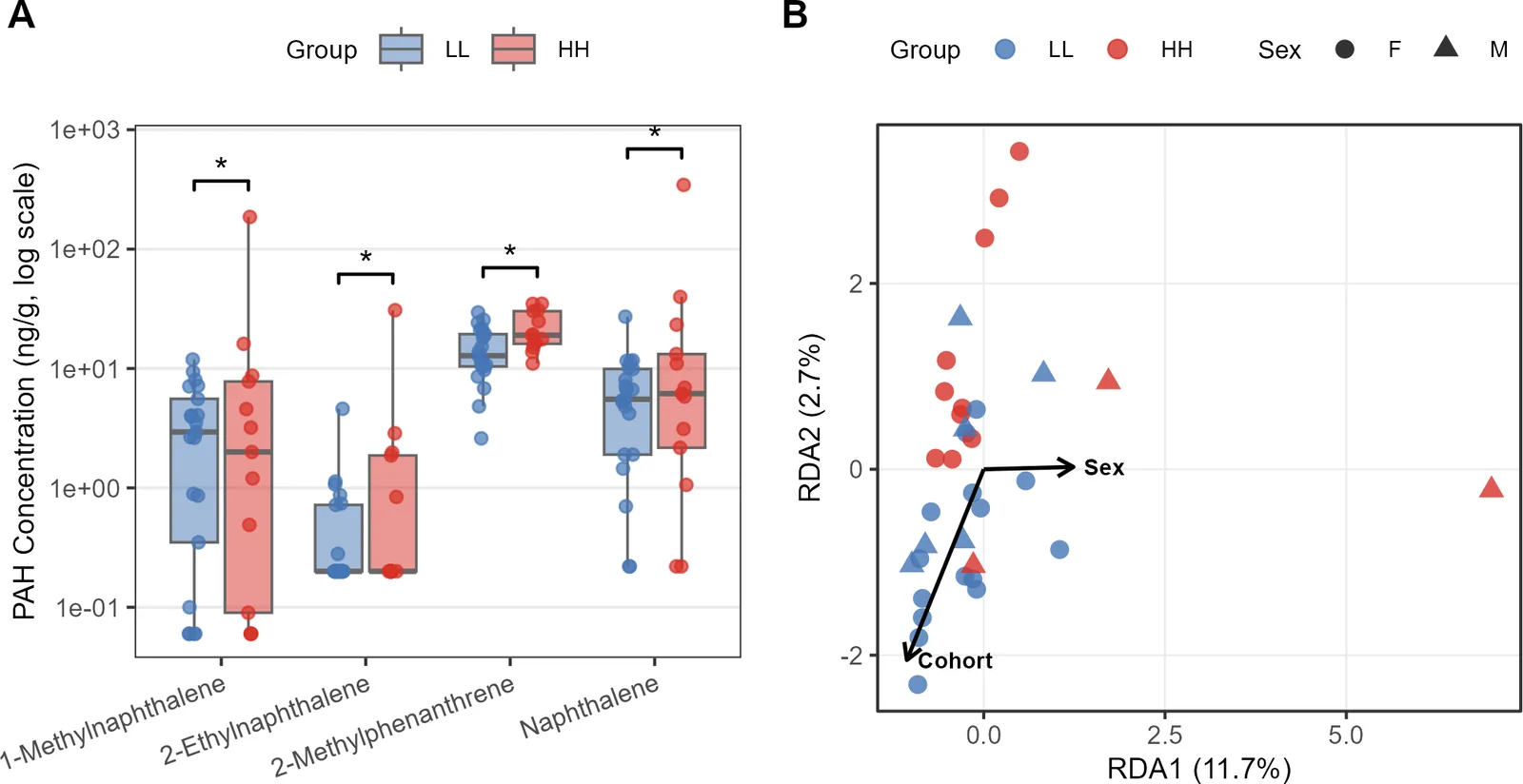
PAH exposure differs by neurobehavioral group and sex. (**A**) PAH concentrations (ng/g, log scale) for the four compounds significantly associated with the neurobehavioral group (negative binomial generalized linear model, FDR < 0.1). HH participants (high impulsivity/poor attention, red) exhibited elevated levels of naphthalene, 1-methylnaphthalene, 2-ethylnaphthalene, and 2-methylphenanthrene relative to LL participants (low impulsivity/fast attention, blue). Asterisks denote FDR < 0.1. (**B**) Redundancy analysis (RDA) of overall PAH exposure profiles constrained by neurobehavioral group and sex. Points represent individual participants colored by group (LL, blue; HH, red) and shaped by sex (circle, female; triangle, male). Vectors indicate the direction and relative magnitude of cohort (HH/LL) and sex effects. A significant interaction between sex and neurobehavioral group predicted PAH exposure composition (PERMANOVA, *P* = 0.026).

Previous work has shown that exposure to PAHs and other xenobiotics is correlated with behaviors that are related to the cognitive trait of impulsivity and attention problems (13, 16, 17). We explored the hypothesis that PAH exposure similarly links to impulsivity and attention in the EMC2 cohort. Our results uncover a significant association between neurobehavioral group membership and exposure to specific PAHs. Individuals from the HH group exhibited markedly elevated levels of four PAHs: naphthalene (*q* = 0.009), 2-ethylnaphthalene (*q* = 0.035), 1-methylnaphthalene (*q* = 0.035), and 2-methylphenanthrene (*q* = 0.067; GLM; Table S4 and Fig. 2A). No PAHs were enriched in the LL group.

Variation in the metabolism of PAHs has been associated with factors ranging from host-genomic capacity to the metabolic activity of the gut microbiome, and it has been shown that PAH detoxification may result in increased production of estrogenic metabolites (28) and sex-dependent factors that influence PAH detoxification (18, 19, 65, 66). Sex-dependent differences are reported for impulsivity (67–69); therefore, a redundancy analysis explored the potential relationship between sex and the overall impulsivity-PAH connection. A significant interaction between sex and neurobehavioral group predicted PAH exposure (PERMANOVA, *P* = 0.026, Fig. 2B). Males exhibited higher PAH levels than females, with this disparity most pronounced in HH males. This finding suggests a potential sex-dependent modulation of the neurobehavioral-PAH association and may reflect underlying biological or behavioral mechanisms that differentially regulate a participant’s exposure to PAHs.

### Gut microbiome diversity is linked to neurobehavioral group and PAH exposure

Variation in the microbiome has been linked to exposure to specific PAHs in prior work (28, 35, 36); therefore, we sought to determine if the composition of the gut microbiome links to the overall PAH exposure profile an individual experiences. Using a Mantel correlation test to examine this relationship, we find a positive correlation between PAH exposure distance (Euclidean distance) and Bray–Curtis microbiome dissimilarity (Mantel test; *r* = 0.22, *P* = 0.032). Moreover, a linear model that uses pairwise Euclidean distances between subjects based on PAH exposure to predict pairwise Bray–Curtis dissimilarity suggests a positive correlation between these metrics of PAH exposure and microbiome diversity (linear model, slope = 8.75, *P* < 0.0001, *R*² = 0.043; Fig. S2). Collectively, these results highlight that variability in PAH exposure tracks with variability in microbiome composition, underscoring the potential for exposure profiles to contribute to microbiome individuality.

**Fig 3:**
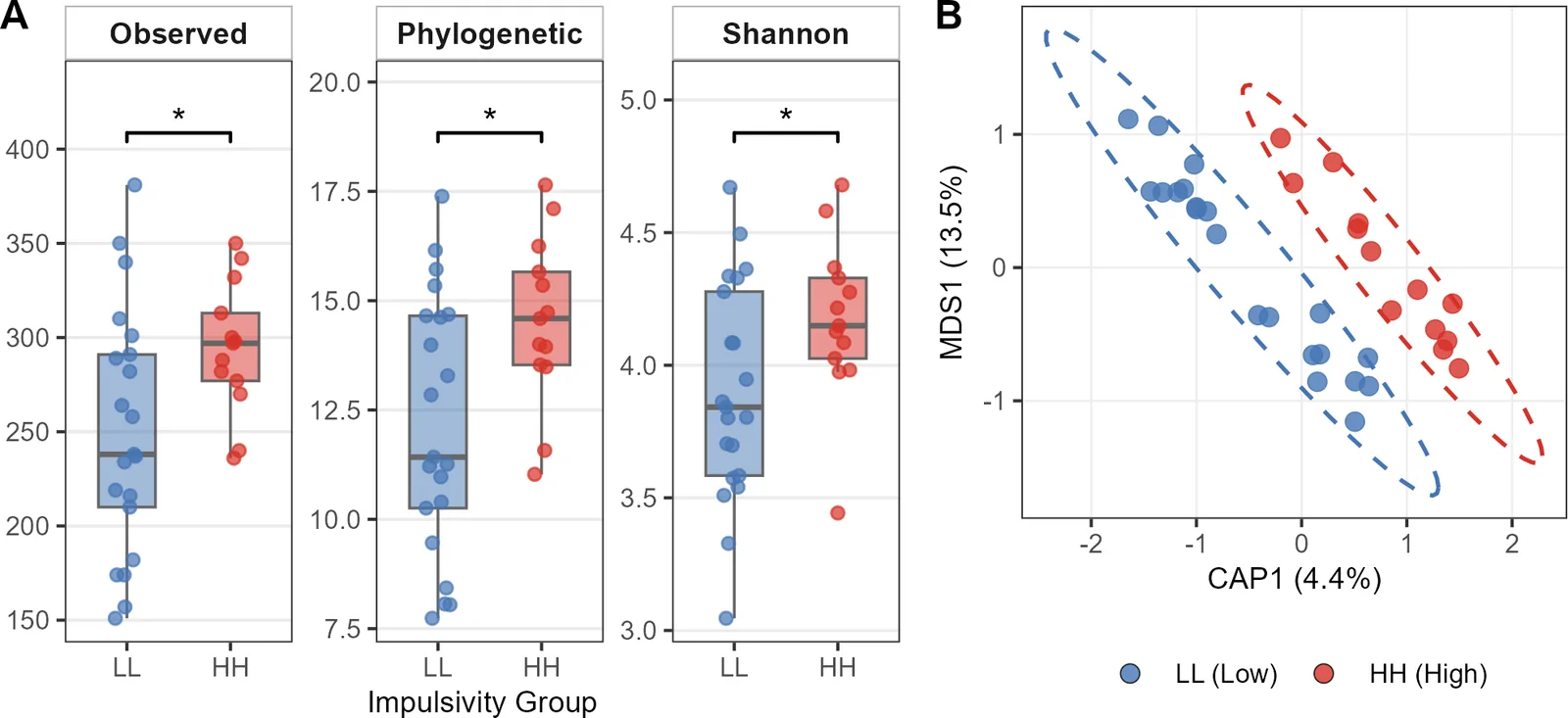
Gut microbiome diversity differs by neurobehavioral group. (**A**) Alpha-diversity metrics—observed taxonomic richness, Faith’s phylogenetic diversity, and Shannon entropy—compared between LL (blue) and HH (red) participants. HH group membership was associated with significantly higher values for all three metrics (generalized linear model; observed richness *P* = 0.033, phylogenetic diversity *P* = 0.016, Shannon *P* = 0.048). Asterisks denote *P* < 0.05. (**B**) Constrained ordination (capscale) of gut microbiome composition using unweighted UniFrac distances. Points represent individual participants colored by neurobehavioral group, with dashed ellipses indicating group dispersion. The neurobehavioral group was significantly associated with community composition (capscale ANOVA, *P* = 0.038).

We next sought to determine if variation in the gut microbiome was also associated with neurobehavioral group membership. We evaluated this relationship using linear models and found that participants in the HH group possessed microbiomes that were more taxonomically and phylogenetically diverse. Specifically, HH group membership was statistically associated with larger levels of microbiome taxonomic richness and Shannon entropy, as well as phylogenetic diversity (generalized linear model; observed richness *P* = 0.033, Shannon *P* = 0.048, Faith’s PD *P* = 0.016; Fig. 3A). We also evaluated how the overall composition of the gut microbiome was associated with the neurobehavioral group, finding a statistically significant relationship between microbiome unweighted-UniFrac distance and HH/LL membership (capscale ANOVA, *P* = 0.038; Fig. 3B). Collectively, these results indicate that the gut microbiome correlates with neurobehavioral phenotype at the community level, and points to a relationship between PAH exposure, neurobehavioral function, and the gut microbiome that warrants further investigation.

### Specific microbial genera are associated with PAH exposure

**Fig 4:**
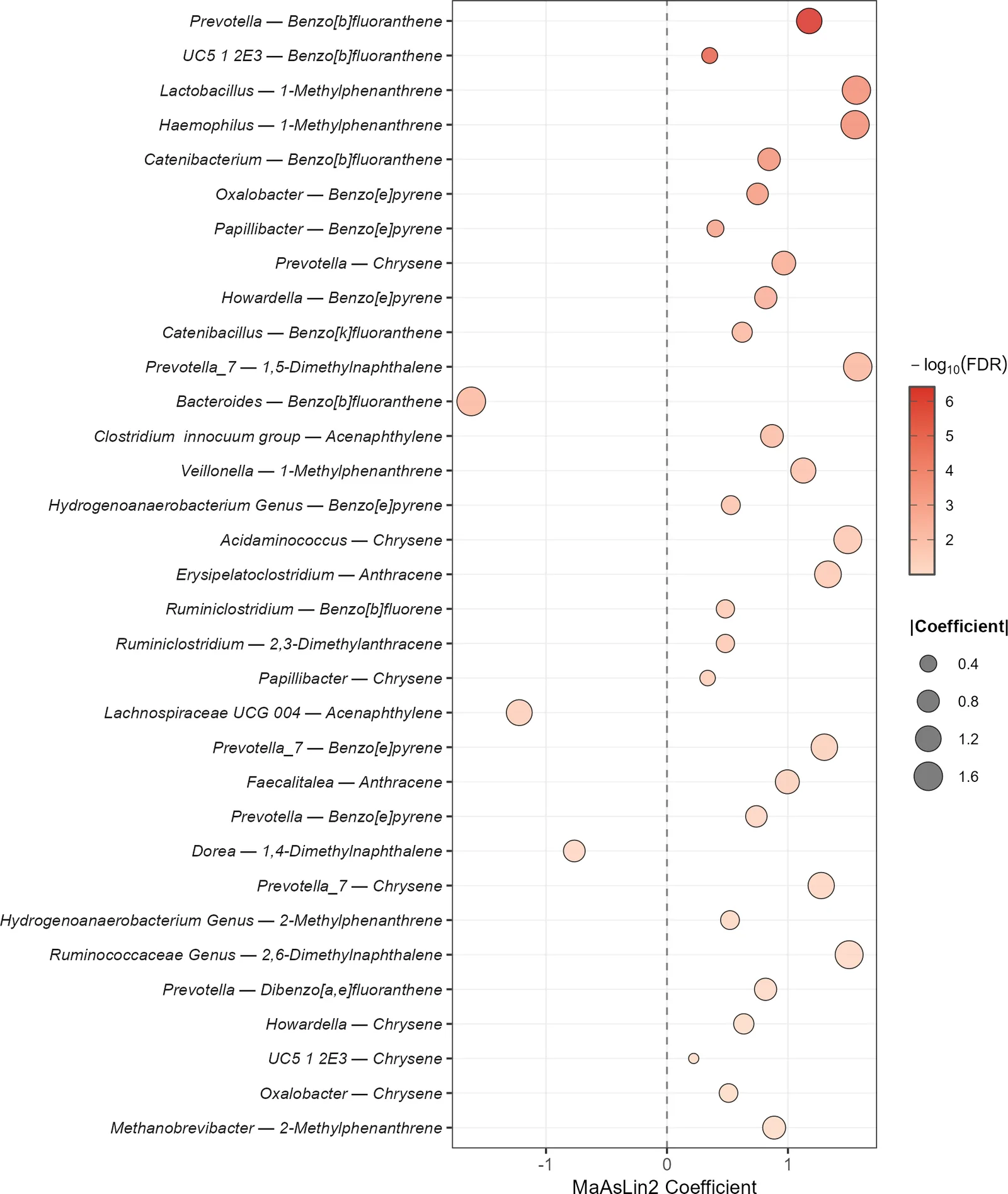
Genus-level associations with PAH exposure identified by MaAsLin2. Each point represents a significant association (FDR < 0.1) between a microbial genus and an individual PAH compound. The *x*-axis shows the MaAsLin2 coefficient (positive values indicate increased genus abundance with higher PAH exposure; negative values indicate decreased abundance). Point size reflects the absolute value of the coefficient, and color intensity reflects statistical significance (−log_10_ FDR). Of 33 significant associations involving 21 genera and 14 PAH compounds, 30 were positive, indicating that higher PAH exposure generally associated with increased genus-level relative abundance. Three genera—*Bacteroides*, *Dorea*, and *Lachnospiraceae* UCG-004—showed negative associations with PAH exposure.

We next sought to determine if specific taxa may underlie the community-level relationship between PAH exposure and microbiome variation. Sequences were agglomerated to the genus level, and MaAsLin2 was used to test associations between genus-level abundance and individual PAH compound concentrations. Of 5,160 genus–PAH pairs tested, 33 exhibited significant associations after correction for multiple testing (FDR < 0.1), involving 22 unique genera and 14 PAH compounds (Table S5). The strongest association was observed between *Prevotella* and benzo[b]fluoranthene, followed by *UC5-1-2E3* and benzo[b]fluoranthene. *Haemophilus* and *Lactobacillus* both exhibited significant positive associations with 1-methylphenanthrene, while *Oxalobacter* and *Papillibacter* were associated with benzo[e]pyrene. *Erysipelatoclostridium* showed a significant positive association with anthracene, and a *Ruminococcaceae* member was positively associated with 2,6-dimethylnaphthalene. Three genera showed negative associations with PAH exposure: *Bacteroides* with benzo[b]fluoranthene, *Dorea* with 1,4-dimethylnaphthalene, and *Lachnospiraceae* UCG-004 with acenaphthylene, each exhibiting decreased abundance with higher PAH exposure. Overall, 30 of 33 significant associations were positive, indicating that higher PAH exposure generally associated with increased genus-level relative abundance (Fig. 4).

No individual genus reached significance for differential abundance between HH (*n* = 13) and LL (*n* = 21) participants after correction for multiple testing (minimum FDR = 0.48). Among the 172 genera tested, 14 showed nominal associations with the neurobehavioral group (*P* < 0.05; Table S6 and Fig. S3). Thirteen of these were more abundant in HH participants, including *Collinsella* (*P* = 0.007), *Angelakisella* (*P* = 0.008), *Hydrogenoanaerobacterium* (*P* = 0.018), and *Methanobrevibacter* (*P* = 0.031); *Tuzzerella* was the sole genus with higher LL abundance (*P* = 0.028). This directional consistency, with nearly all nominally associated taxa enriched in the HH group, parallels the alpha-diversity finding of greater taxonomic richness in HH participants. The absence of individually significant taxa after multiple testing correction is consistent with the limited statistical power of this pilot study, which affords approximately 1% power to detect medium effect sizes (*d* = 0.5) across 172 tests at *n* = 34. Nevertheless, the 14 nominally associated genera provide a candidate set for cross-referencing with the PAH-responsive taxa identified above.

## DISCUSSION

Prior studies independently link PAH exposure to the gut microbiome (28, 35, 36), PAH exposure to impulsivity and attention (13–17), and the microbiome to neurobehavioral variation (70–73), but these three factors have not previously been examined together in a single human cohort. Here, we demonstrate that PAH exposure, gut microbiome composition, and neurobehavioral function covary within a geographically distributed Oregon population. We observed a consistent association between cumulative PAH exposure and neurobehavioral group, modulated by sex, and both variables were independently linked to shifts in gut microbiome composition at the community level. Overall PAH mixture profiles correlated with microbiome dissimilarity, and neurobehavioral group associated with both alpha- and beta-diversity of the gut microbiome. At the taxon level, 22 microbial genera were significantly associated with specific PAH compounds, and a subset of these overlapped with genera nominally associated with neurobehavioral group membership. Together, these results support a three-way relationship among environmental chemical exposure, gut microbiome composition, and neurobehavioral function, and identify candidate taxa at their intersection for targeted investigation.

The relationship between PAH exposure and neurobehavioral phenotype was moderated by sex, with the disparity between HH and LL groups most pronounced among males. This observation is consistent with prior work showing notable differences in the metabolism of PAHs between males and females (18, 19, 65, 66), and with evidence that cell lines from male and female subjects show extensive differences in the abundance of gene transcripts directly related to PAH exposure (74, 75). Several mechanisms could underlie this sex-dependent relationship. Byproducts of PAH metabolism are known to disrupt endocrine signaling, particularly androgen hormone signaling (76, 77), and PAH compounds and their metabolic byproducts are associated with hormone levels in human studies (19, 78, 79). Changes in hormone signaling can have profound impacts on cognitive function, including impulsivity and attention (80, 81). However, it is important to note that our data establish a statistical interaction between sex and neurobehavioral group in predicting PAH exposure; the mechanistic chain from PAH metabolism through endocrine disruption to behavioral change involves several inferential steps that remain to be tested directly.

Beyond the PAH–behavior link, PAH exposure was also associated with gut microbiome composition at the community level. The significant Mantel correlation between pairwise PAH Euclidean distance and Bray–Curtis microbiome dissimilarity (*r* = 0.22, *P* = 0.032) indicates that participants with more similar PAH exposure profiles tend to harbor more similar gut communities. While the effect size is modest (*R*² = 0.043 in the complementary linear model), it is notable in a cohort of this size and consistent with the hypothesis that PAH mixtures—not just individual compounds—exert selective pressure on gut community composition. This finding aligns with animal model studies demonstrating that PAH exposure reshapes overall gut community structure (33–36) and extends these observations to a free-living human population experiencing environmentally relevant, real-world exposure levels.

Our data also point to a relationship between neurobehavioral group and gut microbiome composition that may itself be linked to PAH exposure. This relationship could run in either direction. The HH and LL communities differed less in their dominant members than in their rare ones: HH participants showed greater taxonomic and phylogenetic richness and a distinct composition by unweighted UniFrac distance, a metric sensitive to rare lineages. Behaviors associated with impulsivity offer one explanation for this pattern. Smoking (82), excess alcohol consumption (83), and increased consumption of carbohydrates (84) each reshape the gut microbiome, and smoking and alcohol consumption also alter immune homeostasis (85, 86), which may in turn influence the community by dysregulating immune recognition of commensal taxa and permitting the proliferation of less common lineages. Because smoking is also a major source of PAH exposure (87), these behaviors could contribute to both the elevated PAH burden and the microbiome differences we observe in the HH group. Whether attention-related behaviors have a similar effect is less well characterized.

Conversely, differences in the composition of the gut microbiome may contribute to variation in impulsivity and attention. Gut microbiome composition is an established driver of serotonin physiology (88–91), and serotonin is a suggested modulator of impulsivity, including sex-dependent differences (92). Gut microbes also produce a range of neuroactive metabolites, including short-chain fatty acids and neurotransmitter precursors, that influence neural function through immune, endocrine, and vagal pathways (40–42). Consistent with this direction, gut microbiome composition has been linked to inattention and impulsivity in human case–control studies (93, 94), and preclinical models show that fecal microbiota transplantation can induce or ameliorate attention- and impulsivity-related behaviors (95, 96). These microbiome–brain pathways suggest at least two routes through which PAH exposure could alter neurobehavioral function via the gut microbiome. First, PAH-induced disruption of microbial community structure could destabilize serotonin-related host–microbe interactions, indirectly altering serotonergic signaling to the brain. Second, microbial biotransformation of PAHs could generate estrogenic metabolites that differentially affect males and females, producing sex-divergent behavioral outcomes. This route is supported by evidence that human intestinal microbiota convert PAHs into potent estrogen receptor activators (28) and is consistent with the sex-dependent PAH-neurobehavioral relationship observed in this cohort. Collectively, the behavior–microbiome associations observed in this study are correlational, and we cannot establish their direction or rule out unmeasured differences between the groups. Whether the greater diversity in HH individuals is incidental, reflects a more resilient or protective community, or marks greater environmental or dietary exposure cannot be determined from these cross-sectional data. Distinguishing between these pathways and between them and the behavioral route above will require metabolomic profiling of PAH-derived microbial metabolites in stool, coupled with characterization of circulating estrogen receptor agonist concentrations and serotonergic markers, in cohorts large enough to stratify by sex.

Among the 22 PAH-responsive genera, several have established biological roles that provide context for their sensitivity to PAH exposure. *Prevotella*, associated with high-fiber, plant-rich diets and carbohydrate fermentation (97, 98), showed significant positive associations with benzo[b]fluoranthene, benzo[e]pyrene, chrysene, and dibenzo[a,e]fluoranthene, while *Prevotella_7*, a phylogenetically distinct clade in the SILVA taxonomy, was independently associated with benzo[e]pyrene, chrysene, and 1,5-dimethylnaphthalene. *Lactobacillus*, which showed a significant association with 1-methylphenanthrene, has well-characterized effects on gut–brain axis signaling, including modulation of stress responses and neurotransmitter precursor production (99). Three genera showed negative PAH associations, including *Bacteroides* (benzo[b]fluoranthene, FDR = 0.040), *Dorea* (1,4-dimethylnaphthalene), and *Lachnospiraceae* UCG-004 (acenaphthylene). *Bacteroides* is a dominant commensal whose depletion with PAH exposure could have functional consequences, given its central roles in polysaccharide metabolism and immune regulation (100). Whether these compositional shifts are beneficial or harmful cannot be resolved here: some PAH-associated taxa (e.g., *Haemophilus*) include potential pathobionts, whereas others (e.g., *Lactobacillus*) are generally regarded as beneficial. Because genus-level 16S data cannot resolve pathogenic species or strains, whether PAH-induced shifts favor the expansion of pathobionts is an important question for future work. While these taxon-level associations establish which genera respond to individual PAH compounds, a key question is whether any of these PAH-responsive taxa also track with neurobehavioral phenotype.

Individual taxa did not reach significance for differential abundance between neurobehavioral groups after multiple testing correction, consistent with the limited statistical power of this pilot cohort. However, a central question motivating this study is whether specific gut taxa sit at the intersection of PAH exposure and neurobehavioral phenotype. Cross-referencing the 22 genera with significant PAH associations (FDR < 0.1) against the 14 genera with nominal neurobehavioral associations (*P* < 0.05) identified two genera with directionally coherent associations across both domains. *Hydrogenoanaerobacterium* showed significant positive associations with benzo[e]pyrene (FDR = 0.013) and 2-methylphenanthrene (FDR = 0.065) and was nominally enriched in HH participants (*P* = 0.018). *Methanobrevibacter* showed a significant positive association with 2-methylphenanthrene (FDR = 0.099) and was nominally enriched in HH participants (*P* = 0.031). For both genera, higher PAH exposure and HH group membership are associated with greater abundance. We emphasize that these intersection genera are nominated on the basis of nominal neurobehavioral associations, consistent with the limited power of the group-level analysis noted above, and are therefore hypothesis-generating candidates for targeted study rather than a demonstrated interaction between PAH exposure and neurobehavioral phenotype. Independent literature supports both as biologically plausible candidates. *Hydrogenoanaerobacterium* abundance has been negatively correlated with memory scores in mild cognitive impairment patients (101) and linked to Parkinson’s disease (102). *Methanobrevibacter* is enriched in Parkinson’s disease patients with impulse control disorders, with concurrent enrichment of xenobiotic degradation pathways (102), and is associated with cognitive impairment severity in aged care populations (103) and neuroinflammatory signaling in multiple sclerosis (104, 105). Targeted studies of these candidates in larger cohorts, especially those that incorporate longitudinal designs, metagenomic and metabolomic profiling, and temporal ordering of exposure, microbiome change, and behavioral outcomes, represent a direct path toward testing whether the microbiome mediates the relationship between environmental PAH exposure and neurobehavioral phenotypes.

As a pilot study, this work establishes the empirical basis for several targeted extensions. The modest sample size (13 HH and 21 LL) limits statistical power and the generalizability of these findings, and the associations reported here should be regarded as preliminary and requiring confirmation in larger cohorts. We further note that factors known to shape the gut microbiome, including diet, long-term residence and travel history, comorbidities, and allergies, were not captured beyond our exclusion of recent antibiotic use and could contribute to the community-level variation we observe; the covariates we did measure (age and sex) did not differ between groups (Table S1). Larger cohorts with complete paired data will be needed to move beyond the community-level associations detected here (*n* = 34) and to robustly identify genus-level associations across the full taxonomic breadth after multiple testing correction. Longitudinal sampling designs can address what the present cross-sectional design cannot: the temporal ordering of PAH exposure, microbiome change, and behavioral outcomes necessary to evaluate causal directionality. Our 16S rRNA amplicon approach characterizes which taxa respond to PAH exposure, but metagenomic and metabolomic profiling will be essential to determine whether these taxa are actively metabolizing PAHs or producing neuroactive metabolites. We emphasize that these data establish taxonomic association, not function: they cannot demonstrate that the taxa identified here actively biotransform PAHs or produce neuroactive metabolites. Similarly, future work should pair the 30-day wristband window used here with complementary exposure measures, such as urinary PAH metabolites (recent internal dose) and hair (longer-term exposure), together with repeated microbiome sampling, to clarify whether the associations we observe reflect acute responses or stable, exposure-shaped community states. Together, these extensions would provide the multi-omic depth and temporal resolution needed to test whether the gut microbiome mediates the link between environmental PAH exposure and neurobehavioral outcomes.

In this pilot cohort of 34 adults, PAH exposure, gut microbiome composition, and neurobehavioral function covaried within a single human population. Four PAHs (naphthalene, 1-methylnaphthalene, 2-ethylnaphthalene, and 2-methylphenanthrene) were elevated in the HH group, and this exposure–behavior relationship was modulated by a sex-by-group interaction, with the largest elevations in HH males. HH membership was further associated with greater alpha-diversity and with altered community composition (unweighted UniFrac), and overall PAH exposure profiles associated with microbiome dissimilarity. At the taxon level, we identified significant genus–PAH associations and, by cross-referencing these against genera nominally associated with neurobehavioral group, nominated two hypothesis-generating candidates*—Hydrogenoanaerobacterium* and *Methanobrevibacter*—for targeted study. Because these findings are cross-sectional and correlational, they establish an empirical foundation, rather than causal evidence, for testing whether the gut microbiome mediates the link between PAH exposure and neurobehavioral outcomes.

## Data Availability

The data underlying the findings of this study are available in the NCBI Sequence Read Archive under BioProject ID PRJNA1344322.

## SUPPLEMENTAL MATERIAL

Supplemental tables and figures spectrum.00783-26-s0001.docx Tables S1 to S6 and Fig. S1 to S3.

ASM does not own the copyrights to Supplemental Material that may be linked to, or accessed through, an article. The authors have granted ASM a non-exclusive, world-wide license to publish the Supplemental Material files. Please contact the corresponding author directly for reuse.
