# Supplementary material for: Polycyclic aromatic hydrocarbons, gut microbiome composition, impulsivity, and attention covary in a human cohort
Source: Microbiol Spectr. 2026 Sep 21;14(11):e00783-26. doi: 10.1128/spectrum.00783-26 (PMC13729226; doi:10.1128/spectrum.00783-26)
Supplement: Supplementary file 1 [file spectrum.00783-26-s0001.docx]

**Supplemental Material**

*Polycyclic aromatic hydrocarbons, gut microbiome composition, impulsivity, and attention covary in a human cohort*

Manuscript Spectrum00783-26

**TABLE S1.** Demographic and sample characteristics of EMC2 participants with complete paired data (n = 34). Participants were classified into HH (high impulsivity/poor attention; n = 13) and LL (low impulsivity/fast attention; n = 21) neurobehavioral groups. Kit transit time refers to the number of days between kit delivery to the participant and return via mail. P-values are from two-sample t-tests (continuous variables) or Fisher's exact test (sex). Groups did not differ significantly in age, sex distribution, kit transit time, or 16S rRNA sequencing depth.

| **Variable** | **All** | **HH** | **LL** | **P_value** |
| --- | --- | --- | --- | --- |
| N | 34 | 13 | 21 |  |
| Age, mean (SD) | 41.9 (11.2) | 44.3 (10.0) | 40.4 (11.9) | 0.316 |
| Age, range | 25-71 | 25-59 | 25-71 |  |
| Sex, Female (%) | 25 (74%) | 10 (77%) | 15 (71%) | 1.000 |
| Sex, Male (%) | 9 (26%) | 3 (23%) | 6 (29%) |  |
| Transit time (days), mean (SD) | 45.3 (16.7) | 43.2 (10.2) | 46.7 (20.3) | 0.552 |
| Transit time (days), median [IQR] | 40.0 [33.0-53.0] | 40.0 [38.2-48.5] | 40.0 [33.0-53.0] |  |
| Sequencing depth, mean (SD) | 33226 (20828) | 31476 (17678) | 34309 (22914) | 0.689 |

**TABLE S2.** Cross-tabulation of the EMC2 recruitment pool by impulsivity quartile (BIS-15 total score) and attention quartile (PVT-B median reaction time) among 2,628 participants who completed both assessments. Highlighted cells denote the LL recruitment group (Q1 impulsivity × Q1 attention; low impulsivity, fast reaction times) and the HH recruitment group (Q4 impulsivity × Q4 attention; high impulsivity, slow reaction times). Group sizes did not differ significantly (Chi-square p = 0.33).

| **Impulsivity Quartile** | **Q1 Attention (fast)** | **Q2 Attention** | **Q3 Attention** | **Q4 Attention (slow)** | **Total** |
| --- | --- | --- | --- | --- | --- |
| Q1 (low) | 291 (44%) | 250 (38%) | 272 (42%) | 272 (41%) | 1,085 (41%) |
| Q2 | 159 (24%) | 143 (22%) | 149 (23%) | 143 (22%) | 594 (23%) |
| Q3 | 91 (14%) | 110 (17%) | 90 (14%) | 101 (15%) | 392 (15%) |
| Q4 (high) | 126 (19%) | 157 (24%) | 133 (21%) | 141 (22%) | 557 (21%) |
| Total | 667 (100%) | 660 (100%) | 644 (100%) | 657 (100%) | 2,628 (100%) |

*Shaded cells denote the LL (Q1 × Q1) and HH (Q4 × Q4) recruitment groups.*

**TABLE S3.** Attention metrics differ between lowest (Q1) and highest (Q4) impulsivity quartiles. Independent-samples t-tests comparing median reaction time, lapses (errors of omission), and false starts (errors of commission) between Q1 (n = 283) and Q4 (n = 139) participants. All metrics differed significantly (p < 0.005). Levene's test indicated unequal variances for all metrics (p < 0.002); degrees of freedom and test statistics reflect Welch's correction.

| **Metric** | **Q1 (low) n=283 Mean** | **Q1 (low) n=283 SD** | **Q4 (high) n=139 Mean** | **Q4 (high) n=139 SD** | **Test Statistic** | **p-value** |
| --- | --- | --- | --- | --- | --- | --- |
| Median Reaction Time (msec) | 296.56 | 13.70 | 511.65 | 896.45 | t(138)=-2.83 | 0.005 |
| Lapses | 8.55 | 4.16 | 46.27 | 11.34 | t(156)=-37.98 | <0.001 |
| False Starts | 3.06 | 4.82 | 1.36 | 1.95 | t(408)=5.14 | <0.001 |

**TABLE S4.** Negative binomial generalized linear model results for PAH compound concentrations as a function of neurobehavioral group (HH vs. LL). Each of 30 PAH compounds detected in silicone wristbands was modeled independently. Columns show group means (ng/g), model estimate, standard error, z-statistic, uncorrected p-value, Benjamini-Hochberg FDR-adjusted q-value, and direction of enrichment. Four PAHs were significantly elevated in HH participants after multiple testing correction (FDR < 0.1): naphthalene, 1-methylnaphthalene, 2-ethylnaphthalene, and 2-methylphenanthrene. No PAHs were significantly enriched in the LL group. Results are sorted by FDR.

| **PAH** | **Mean_HH** | **Mean_LL** | **Estimate** | **SE** | **z** | **P_value** | **FDR** | **Direction** |
| --- | --- | --- | --- | --- | --- | --- | --- | --- |
| Naphthalene | 35.23 | 6.77 | -1.6491 | 0.4561 | -3.616 | 0.000299 | 0.00898 | Higher in HH |
| 1-Methylnaphthalene | 17.72 | 3.59 | -1.596 | 0.5467 | -2.919 | 0.00351 | 0.0351 | Higher in HH |
| 2-Ethylnaphthalene | 3.07 | 0.58 | -1.6652 | 0.5585 | -2.981 | 0.00287 | 0.0351 | Higher in HH |
| 2-Methylphenanthrene | 21.85 | 14.66 | -0.3991 | 0.1527 | -2.613 | 0.00898 | 0.0674 | Higher in HH |
| 1,2-Dimethylnaphthalene | 2.12 | 0.55 | -1.3557 | 0.606 | -2.237 | 0.0253 | 0.152 | Higher in HH |
| Benzo[e]pyrene | 0.68 | 0.15 | -1.5137 | 0.7377 | -2.052 | 0.0402 | 0.201 | Higher in HH |
| 2,3-Dimethylanthracene | 0.46 | 0.07 | -1.8911 | 1.0026 | -1.886 | 0.0593 | 0.254 | Higher in HH |
| Fluoranthene | 13.47 | 9.41 | -0.3586 | 0.2078 | -1.725 | 0.0844 | 0.317 | Higher in HH |
| Anthracene | 1.35 | 3.34 | 0.9066 | 0.5755 | 1.575 | 0.115 | 0.346 | Higher in LL |
| Phenanthrene | 41.27 | 32.02 | -0.2538 | 0.1565 | -1.622 | 0.105 | 0.346 | Higher in HH |
| Benzo[a]fluorene | 0.72 | 0.35 | -0.7192 | 0.4931 | -1.459 | 0.145 | 0.395 | Higher in HH |
| 2,6-Dimethylnaphthalene | 1.55 | 3.51 | 0.8158 | 0.5957 | 1.369 | 0.171 | 0.427 | Higher in LL |
| 2-Methylanthracene | 15.56 | 10.18 | -0.4239 | 0.3352 | -1.264 | 0.206 | 0.469 | Higher in HH |
| 2-Methylnaphthalene | 6.66 | 8.85 | 0.2839 | 0.2457 | 1.155 | 0.248 | 0.469 | Higher in LL |
| Dibenzothiophene | 4.19 | 3.36 | -0.2187 | 0.1902 | -1.15 | 0.25 | 0.469 | Higher in HH |
| Retene | 16.12 | 13.43 | -0.1828 | 0.1535 | -1.191 | 0.234 | 0.469 | Higher in HH |
| 1,4-Dimethylnaphthalene | 0.99 | 0.58 | -0.5372 | 0.5326 | -1.009 | 0.313 | 0.494 | Higher in HH |
| 3,6-Dimethylphenanthrene | 4.27 | 2.86 | -0.401 | 0.3897 | -1.029 | 0.303 | 0.494 | Higher in HH |
| Benzo[k]fluoranthene | 0.26 | 0.58 | 0.8119 | 0.7762 | 1.046 | 0.296 | 0.494 | Higher in LL |
| Benzo[b]fluorene | 0.57 | 0.35 | -0.485 | 0.5209 | -0.931 | 0.352 | 0.528 | Higher in HH |
| 1,5-Dimethylnaphthalene | 0.98 | 0.71 | -0.3251 | 0.5172 | -0.629 | 0.53 | 0.667 | Higher in HH |
| Acenaphthylene | 0.82 | 1.06 | 0.2617 | 0.4207 | 0.622 | 0.534 | 0.667 | Higher in LL |
| Dibenzo[a,e]fluoranthene | 0.1 | 0.2 | 0.6763 | 1.0057 | 0.673 | 0.501 | 0.667 | Higher in LL |
| Triphenylene | 0.6 | 0.39 | -0.4334 | 0.6651 | -0.652 | 0.515 | 0.667 | Higher in HH |
| 1-Methylphenanthrene | 1.81 | 2.43 | 0.2957 | 0.6638 | 0.446 | 0.656 | 0.705 | Higher in LL |
| 1-Methylpyrene | 0.67 | 0.86 | 0.2495 | 0.527 | 0.473 | 0.636 | 0.705 | Higher in LL |
| Benzo[b]fluoranthene | 0.08 | 0.14 | 0.5765 | 1.1384 | 0.506 | 0.613 | 0.705 | Higher in LL |
| Fluorene | 8.91 | 10.71 | 0.1834 | 0.4145 | 0.442 | 0.658 | 0.705 | Higher in LL |
| Benz[a]anthracene | 0.54 | 0.42 | -0.2393 | 0.6087 | -0.393 | 0.694 | 0.718 | Higher in HH |
| Chrysene | 0.58 | 0.51 | -0.1321 | 0.6158 | -0.215 | 0.83 | 0.83 | Higher in HH |

**TABLE S5.** MaAsLin2 results for all significant genus–PAH associations (FDR < 0.1). All 33 significant associations involving 22 unique genera and 14 PAH compounds are listed with effect estimates, standard errors, p-values, and FDR-adjusted q-values. Genus-level abundances were TSS-normalized and log-transformed prior to model fitting.

| **Genus** | **PAH** | **Coefficient** | **SE** | **N** | **N_nonzero** | **P_value** | **FDR** |
| --- | --- | --- | --- | --- | --- | --- | --- |
| Prevotella | Benzo[b]fluoranthene | 1.176 | 0.143 | 34 | 4 | 2.27e-09 | 3.91e-07 |
| UC5 1 2E3 | Benzo[b]fluoranthene | 0.353 | 0.051 | 34 | 4 | 9.06e-08 | 7.79e-06 |
| Lactobacillus | 1-Methylphenanthrene | 1.566 | 0.272 | 34 | 7 | 2.16e-06 | 0.000186 |
| Haemophilus | 1-Methylphenanthrene | 1.555 | 0.267 | 34 | 12 | 1.85e-06 | 0.000186 |
| Catenibacterium | Benzo[b]fluoranthene | 0.844 | 0.153 | 34 | 4 | 4.51e-06 | 0.000259 |
| Oxalobacter | Benzo[e]pyrene | 0.749 | 0.132 | 34 | 7 | 2.9e-06 | 0.000498 |
| Papillibacter | Benzo[e]pyrene | 0.401 | 0.077 | 34 | 9 | 1.18e-05 | 0.00102 |
| Prevotella | Chrysene | 0.966 | 0.186 | 34 | 4 | 1.14e-05 | 0.00197 |
| Howardella | Benzo[e]pyrene | 0.817 | 0.172 | 34 | 8 | 4.04e-05 | 0.00231 |
| Catenibacillus | Benzo[k]fluoranthene | 0.622 | 0.127 | 34 | 5 | 2.7e-05 | 0.00464 |
| Prevotella_7 | 1,5-Dimethylnaphthalene | 1.577 | 0.323 | 34 | 7 | 2.81e-05 | 0.00483 |
| Bacteroides | Benzo[b]fluoranthene | -1.618 | 0.369 | 34 | 33 | 0.000117 | 0.00504 |
| Clostridium innocuum group | Acenaphthylene | 0.868 | 0.183 | 34 | 12 | 4.06e-05 | 0.00699 |
| Veillonella | 1-Methylphenanthrene | 1.127 | 0.268 | 34 | 11 | 2e-04 | 0.0115 |
| Hydrogenoanaerobacterium Genus | Benzo[e]pyrene | 0.528 | 0.13 | 34 | 5 | 0.000302 | 0.013 |
| Acidaminococcus | Chrysene | 1.495 | 0.359 | 34 | 11 | 0.000219 | 0.0188 |
| Erysipelatoclostridium | Anthracene | 1.331 | 0.305 | 34 | 24 | 0.000122 | 0.021 |
| Ruminiclostridium | Benzo[b]fluorene | 0.483 | 0.111 | 34 | 4 | 0.000123 | 0.0211 |
| Ruminiclostridium | 2,3-Dimethylanthracene | 0.483 | 0.111 | 34 | 4 | 0.000123 | 0.0211 |
| Papillibacter | Chrysene | 0.336 | 0.086 | 34 | 9 | 0.000476 | 0.0273 |
| Lachnospiraceae UCG 004 | Acenaphthylene | -1.221 | 0.307 | 34 | 30 | 0.000373 | 0.0321 |
| Prevotella_7 | Benzo[e]pyrene | 1.301 | 0.359 | 34 | 7 | 0.001 | 0.0345 |
| Faecalitalea | Anthracene | 0.995 | 0.253 | 34 | 15 | 0.000423 | 0.0363 |
| Prevotella | Benzo[e]pyrene | 0.739 | 0.216 | 34 | 4 | 0.00173 | 0.0495 |
| Dorea | 1,4-Dimethylnaphthalene | -0.766 | 0.19 | 34 | 34 | 0.000324 | 0.0557 |
| Prevotella_7 | Chrysene | 1.275 | 0.362 | 34 | 7 | 0.00132 | 0.0568 |
| Hydrogenoanaerobacterium Genus | 2-Methylphenanthrene | 0.521 | 0.131 | 34 | 5 | 0.000379 | 0.0652 |
| Ruminococcaceae Genus | 2,6-Dimethylnaphthalene | 1.506 | 0.38 | 34 | 33 | 0.00039 | 0.067 |
| Prevotella | Dibenzo[a,e]fluoranthene | 0.815 | 0.207 | 34 | 4 | 0.000424 | 0.0729 |
| Howardella | Chrysene | 0.635 | 0.194 | 34 | 8 | 0.00259 | 0.0892 |
| UC5 1 2E3 | Chrysene | 0.221 | 0.071 | 34 | 4 | 0.00376 | 0.0987 |
| Oxalobacter | Chrysene | 0.509 | 0.164 | 34 | 7 | 0.00402 | 0.0987 |
| Methanobrevibacter | 2-Methylphenanthrene | 0.886 | 0.248 | 34 | 8 | 0.00116 | 0.0994 |

**TABLE S6.** MaAsLin2 results for genus–neurobehavioral group associations. No genera reached significance after FDR correction (minimum FDR = 0.48). The 14 genera with nominal associations (uncorrected p < 0.05) are shown with effect estimates, standard errors, p-values, and FDR-adjusted q-values. Thirteen of 14 nominally associated genera were more abundant in HH participants.

| **Genus** | **Coefficient** | **SE** | **N** | **N_nonzero** | **P_value** | **FDR** | **Direction** |
| --- | --- | --- | --- | --- | --- | --- | --- |
| Collinsella | -1.981 | 0.689 | 34 | 27 | 0.0071 | 0.48 | Higher in HH |
| NK4A214 group | -2.911 | 1.032 | 34 | 21 | 0.00813 | 0.48 | Higher in HH |
| Angelakisella | -1.139 | 0.405 | 34 | 14 | 0.00838 | 0.48 | Higher in HH |
| Lachnospiraceae UCG-010 | -1.373 | 0.551 | 34 | 30 | 0.018 | 0.577 | Higher in HH |
| Hydrogenoanaerobacterium | -0.74 | 0.298 | 34 | 5 | 0.0182 | 0.577 | Higher in HH |
| UCG-002 | -2.724 | 1.136 | 34 | 26 | 0.0225 | 0.577 | Higher in HH |
| Family XIII AD3011 group | -1.522 | 0.653 | 34 | 25 | 0.0261 | 0.577 | Higher in HH |
| Tuzzerella | 1.729 | 0.75 | 34 | 20 | 0.0277 | 0.577 | Higher in LL |
| Methanobrevibacter | -1.246 | 0.552 | 34 | 8 | 0.0311 | 0.577 | Higher in HH |
| Phocea | -0.668 | 0.301 | 34 | 17 | 0.0335 | 0.577 | Higher in HH |
| Oribacterium | -0.613 | 0.289 | 34 | 5 | 0.0418 | 0.594 | Higher in HH |
| Eubacterium ruminantium group | -1.314 | 0.624 | 34 | 7 | 0.043 | 0.594 | Higher in HH |
| Eggerthellaceae (unclassified) | -0.997 | 0.478 | 34 | 8 | 0.0449 | 0.594 | Higher in HH |
| Paraprevotella | -1.761 | 0.864 | 34 | 9 | 0.0499 | 0.614 | Higher in HH |

**FIG S1.** Participant flow through EMC2 recruitment and selection. Six-stage diagram showing Healthy Oregon Project participants who completed the BIS-15 impulsivity assessment (n = 3,106) and the PVT-B attention assessment (n = 2,678), those who completed both instruments (n = 2,628), those qualifying for LL or HH group assignment after exclusions for missing sex, age, impulsivity, or reaction-time data (n = 422; LL = 283, HH = 139), those invited to participate (n = 50), and those providing complete paired data across all three domains (n = 34; HH = 13, LL = 21).

**FIG S2.** Community-level correlation between PAH exposure and gut microbiome composition. Scatterplot of pairwise Bray-Curtis dissimilarity (x-axis; computed from rarefied 16S rRNA ASV-level abundances) against pairwise Euclidean distance of standardized PAH concentrations (y-axis) for all participant pairs (n = 561 pairwise comparisons). Blue line indicates linear model fit (slope = 8.75, p < 0.0001, R² = 0.043); shaded band indicates 95% confidence interval. The Mantel test confirmed a significant positive correlation between PAH exposure distance and microbiome dissimilarity (Spearman r = 0.22, p = 0.032, 5,000 permutations).

**FIG S3.** Rarefied abundances of the 14 genera with nominal associations (uncorrected p < 0.05) with neurobehavioral group (HH vs. LL), identified by MaAsLin2. Each panel shows one genus, with individual data points overlaid on boxplots for LL (blue) and HH (red) participants. Thirteen of 14 genera were more abundant in HH participants; Tuzzerella was the sole genus with higher LL abundance. No genera reached significance after FDR correction (minimum FDR = 0.48).
